# Control of glucose metabolism is important in tenogenic differentiation of progenitors derived from human injured tendons

**DOI:** 10.1371/journal.pone.0213912

**Published:** 2019-03-18

**Authors:** Soutarou Izumi, Satoru Otsuru, Nobuo Adachi, Ngozi Akabudike, Motomi Enomoto-Iwamoto

**Affiliations:** 1 Department of Orthopaedics, University of Maryland, Baltimore, Baltimore, Maryland, United States of America; 2 Department of Orthopaedic Surgery, Hiroshima University, Hiroshima, Japan; Louisiana State University, UNITED STATES

## Abstract

Glucose metabolism is altered in injured and healing tendons. However, the mechanism by which the glucose metabolism is involved in the pathogenesis of tendon healing process remains unclear. Injured tendons do not completely heal, and often induce fibrous scar and chondroid lesion. Because previous studies have shown that tendon progenitors play roles in tendon repair, we asked whether connective tissue progenitors appearing in injured tendons alter glucose metabolism during tendon healing process. We isolated connective tissue progenitors from the human injured tendons, obtained at the time of primary surgical repair of rupture or laceration. We first characterized the change in glucose metabolism by metabolomics analysis using [1,2-^13^C]-glucose using the cells isolated from the lacerated flexor tendon. The flux of glucose to the glycolysis pathway was increased in the connective tissue progenitors when they proceeded toward tenogenic and chondrogenic differentiation. The influx of glucose to the tricarboxylic acid (TCA) cycle and biosynthesis of amino acids from the intermediates of the TCA cycle were strongly stimulated toward chondrogenic differentiation. When we treated the cultures with 2-deoxy-D-glucose (2DG), an inhibitor of glycolysis, 2DG inhibited chondrogenesis as characterized by accumulation of mucopolysaccharides and expression of *AGGRECAN*. Interestingly, 2DG strongly stimulated expression of tenogenic transcription factor genes, *SCLERAXIS* and *MOHAWK* under both chondrogenic and tenogenic differentiation conditions. The findings suggest that control of glucose metabolism is beneficial for tenogenic differentiation of connective tissue progenitors.

## Introduction

Glucose metabolism, intercrossed with various metabolic pathways, such as biosynthesis of amino acids and nucleotides and lipid metabolism, plays important roles in development and homeostasis of organs and tissues [1, 2]. Alterations of glucose metabolism are found in various pathological conditions including cancer, inflammation and wound healing [3–7]. The importance of glucose metabolism in tendon healing has been demonstrated clinically and experimentally. Increases in glucose uptake and glucose metabolites have been found in human healing Achilles tendons [8, 9]. The similar response has been demonstrated in the mouse Achilles injury model: Acceleration of glycolysis, lactate synthesis and tricarboxylic acid (TCA) cycle were demonstrated in tendons acutely after injury and retained for 4 weeks after injury [10]. The findings indicate that glucose metabolism is greatly altered in injured tendons. Hyperglycemia in diabetes is a risk for tendinopathy, tendon rupture and impaired tendon healing [11–13]. High glucose environment may disturb biochemical, biomechanical and biological function and would be a risk factor for tendon tear [14, 15]. However, the mechanism(s) by which the glucose metabolism is involved in the pathogenesis of tendon healing process remains unclear.

Once injured, a tendon usually does not regain original structure and mechanical strength. The damaged tendon often proceeds toward degenerative processes that include formation of fibrous and vascular scar tissue and accumulation of mucopolysaccharides [16, 17]. These processes can be caused by the cells present in the injured sites [18, 19]. Recently we have found that connective tissue progenitor cells appear in injured tendons and can contribute to tendon healing and chondroid degeneration [20]. Studies have demonstrated that high glucose directly modulates cell function and reduces expression of tendon-related molecules in tendon derived stem cells [21]. Taken together, we hypothesized that progenitors appearing in injured tendons change glucose metabolism during their differentiation and asked this question by performing the metabolomics analysis using [1,2-^13^C]-glucose. Our results demonstrated that the progenitors isolated from human injured tendons stimulated glycolysis and TCA cycle pathway when they proceeded toward chondrogenic differentiation. Furthermore, we found that 2-deoxy-D-glucose (2DG), an inhibitor of glycolysis inhibited chondrogenic differentiation while stimulated gene expression of tenogenic transcription factors, *SCLERAXIS* and *MOHAWK*. The results indicate that control of glucose metabolism could be beneficial for stimulation of tenogenic differentiation and inhibition of chondrogenic differentiation of connective tissue progenitors during tendon healing.

## Materials and methods

### Isolation of human injured tendon-derived connective tissue progenitors (hITPC)

Human subject research, determined as non human subjects by the institutional review at University of Maryland, Baltimore. The injured tendon tissues were obtained from human ruptured Achilles tendons and lacerated flexor tendons in Zone 2 at the time of surgical repair. 2 Achilles tendons (2 males, age 55 and 56) and 5 flexor tendons from 5 patients (5 males, ages 24, 32, 27, 35, 40). The tissue samples were exempt and considered non- human subjects since the specimens were completely de-identified as determined by the Institutional Review Board. The tendon progenitors were isolated according to the methods previously reported [20, 22]. The tendon tissues were digested by incubating with 2.5 unit/ml Dispase and 600 unit/ml Collagenase Type I in Hank’s balanced salt solution (HBSS) for 1h at 37°C with gentle shaking. The dissociated cells were plated on the culture dish at a density of 70–140 cells/cm^2^ in 60-mm dishes and cultured in Dulbecco’s modified eagle medium (high glucose DMEM containing 4.5g/L, Gibco, Gaithersburg, MD) containing 20% of fetal bovine serum (FBS, Gemni Bio-products, West Sacrament, CA). The cultures were expanded and confirmed expression of stem cell markers by flow cytometry. The expanded cells were cryopreserved for all batches. The cultures between passage 3 and 7 were used. The cells isolated from the injured flexor tendons were used for 13C-glucose metabolic analysis and analysis of 2DG actions. The cells isolated from the injured Achilles tendons were used only for analysis of 2DG actions.

### Cultures of hITPCs

The cells isolated from injured flexor tendons and Achilles tendons were plated at the density of 200,000 cells/well in 12-well plate and cultured in monolayer with or without recombinant human bone morphogenetic protein 12 (rhBMP12) (100ng/ml, R&D Systems, Minneapolis, MN) in DMEM containing 10% FBS and 50 μg/ml ascorbic acid for 7 days. For the micromass cultures, the cells were plated at the density of 200,000 cells in 20 μl and fed with DMEM containing 10% FBS and 50 μg/ml ascorbic acid for 7 days. We have previously found that progenitor cells isolated from injured tendons have stronger chondrogenic potential and exhibited chondrogenic phenotype in micromass culture without exogenous chondrogenic factors [20]. The cultures were treated with 2-deoxy-D-glucose (Sigma-Aldrich, St. Louis, MO), or vehicle from Day 1 for 7 days and subjected to the ^13^C-glucose metabolic flux, cytochemical and gene expression analyses.

### ^13^C-glucoce metabolic flux analysis

The cells isolated from injured flexor tendons were incubated with glucose-free DMEM containing 25 mM [1,2-^13^C]glucose (Sigma-Aldrich), supplemented with 10% dialyzed serum (Invitrogen, Carlsbad, CA) for 48 hrs. The cultures were then washed twice with cold PBS and then incubated with 4% perchloric acid (Sigma-Aldrich) for 30 min at 4C on a rocking platform. Cell extracts were neutralized using 5M KOH. After centrifugation, supernatants were subjected to an AG-1 column (100–200 mesh, 0.5x2.5 cm, Bio-rad, Hercules, CA) for enriching the organic acids, glutamate and aspartate, which were then converted to t-butyldimethylsilyl derivatives. For measurement of the ^13^C enrichment, samples were prepared as described previously [23–25]. Isotopic enrichment in [^13^C]-aspartate isotopomers was monitored using ions at m/z 418 and 420 for M0 and M2 containing 2 ^13^C atoms above M0, the natural abundance, respectively. Isotopic enrichment in [^13^C]-lactate was determined using ions atm/z 261 and 263 for M0 and M2 containing 2 ^13^C atoms above natural abundance, respectively. Isotopic enrichment in [^13^C]-malate isotopomers was evaluated using ions at m/z 419 and 42 for M0 and M2 containing 2 ^13^C atoms above natural abundance, respectively, and ^13^C enrichment in [^13^C]-citrate isotopomers was assayed using ions atm/z 459 and 461 for M0 and M2 containing 2 ^13^C atoms above natural abundance, respectively.

Isotopic enrichment in [^13^C]-glucose 6-phosphate, [^13^C]-ribose 5-phosphate and [^13^C]-glyceraldehyde 3-phosphate were determined using a liquid chromatography-mass spectrometry (LC-MS) system as previously described [24]. For ribose in multiple-reaction monitoring (MRM) mode, we measured ion-pairs 561–175 and 562–175 for determination of M0 and M1 containing 1 ^13^C atoms above natural abundance, respectively.

### Flow cytometric analysis

The hITPCs were labeled with Human Mesenchymal Stem Cell Multi-Color Flow kit (R&D Systems, Minneapolis, MN) following the manufacturer’s protocol. The stem cell marker expressions were detected by LSR II flow cytometer (BD, Franklin, NJ) and analyzed with FlowJo (FlowJo, Ashland, OR). This kit contains antibodies against CD90-APC, CD73-CFS and CD105-PerCP, and the cocktail of hematopoietic markers such as CD45-PE, CD34-PE, CD11b-PE, CD79A-PE and HLA-DR-PE.

### Cytochemical analysis

The cultures were fixed with 4% paraformaldehyde and stained with alcian blue (pH 1.0). The staining images were captured using BZ-X710 (Keyence Corporation, Itasca, IL). Using Image J, the integrated density of the staining was measured.

### Gene expression analysis

Total RNAs were extracted from the cells using RNeasy Mini Kit (QIAGEN, Hilden, Germany). The cDNAs were synthesized cDNA with the EcoDry^™^ Premix (Clontech, Mountain View, CA) in a 20μl reaction volume. Quantitative PCR (qPCR) was performed using TaqMan FastAdvanced Master Mix (Applied Biosystems, Foster City, CA). The probes are as follows; *SCX*: Hs03054634_g1, *COL1A1*: Hs00164004_m1, *ACTB*: Hs99999903_m1, *SOX9*: Hs00165814_m1, *MKX*: Hs00543190_m1, *ACAN*: Hs00153936_m1, *TNMD*: Hs00223332_m1. Reactions were performed with 3 biological replicates. Changes in transcript abundance were calculated using the ΔΔCt method with *ACTB* as the reference transcript following the manufacturer protocol.

### Protein assay

The cultures were lysed in Saline containing 0.1% Triton-X and 0.01N NaOH. Proteins were measured using Pierce^™^ BCA Protein Assay Kit (Thermo Scientific, Waltham, MA) following the manufacturer’s protocol.

### ATP assay

The cultures were lysed containing 0.1% Triton-X and 0.01N NaOH. ATP assay was performed using Luminescent ATP Detection Assay Kit (Abcam plc, Cambridge, UK) following the manufacturer’s protocol. All the luminescence was measured with 1 second per well.

### DNA assay

The cultures were lysed containing 0.1% Triton-X and 0.01N NaOH. DNA contents were measured using CyQUANT Cell Proliferation Assay Kit (Thermo Scientific, Waltham, MA) following the manufacturer’s protocol.

### Statistical methods

Results were analyzed by Student’s t test or ordinary one-way ANOVA and Turkey’s multiple comparison test using Prism 6 (GraphPad Software, La Jolla, CA). The threshold for significance for all tests was set as *p* < 0.05.

## Results

### Connective tissue progenitors isolated from human injured tendons

**Fig 1:**
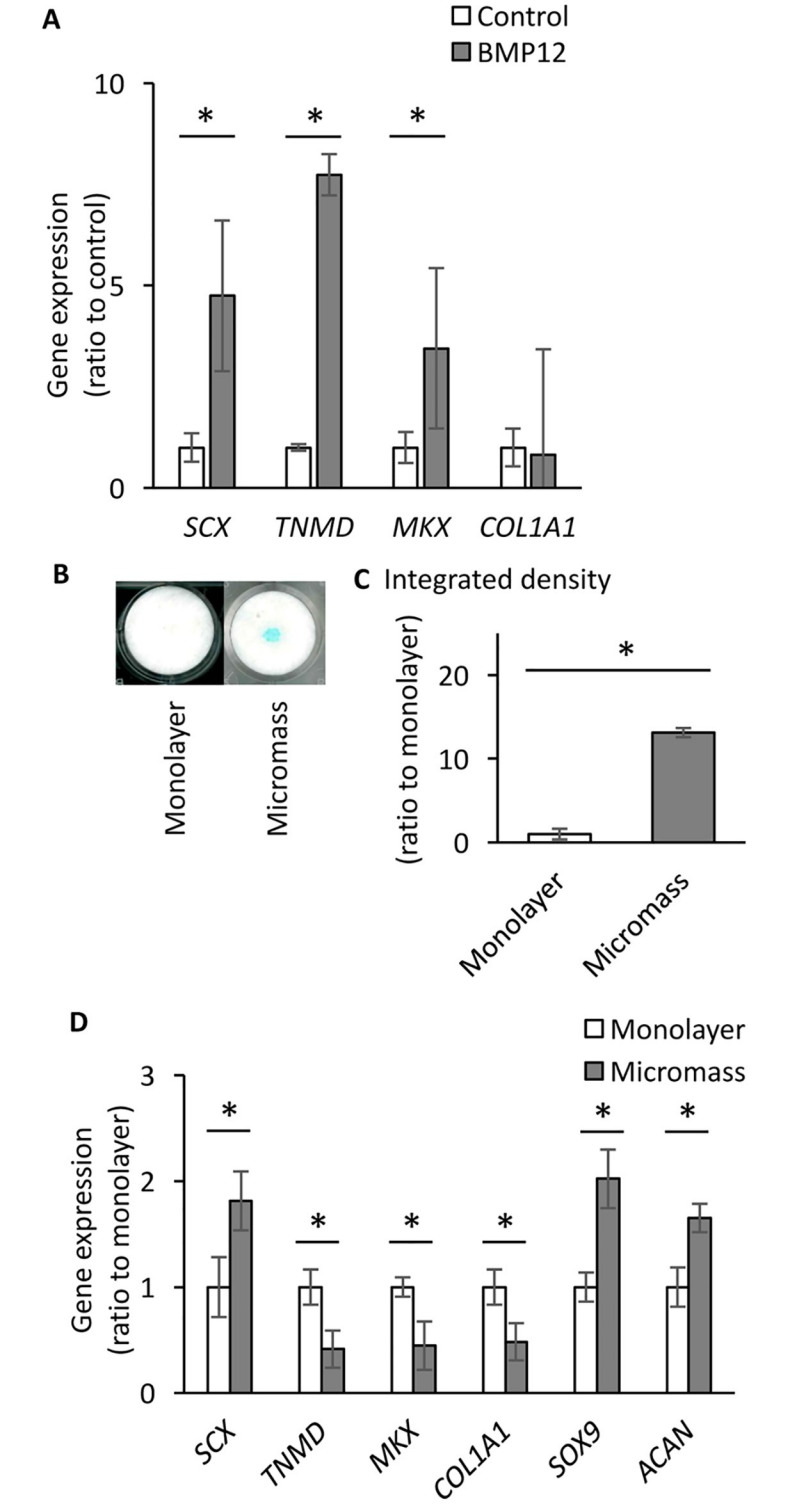
Characterization of hITPCs in monolayer and micromass cultures. (A) Progenitor cells obtained from human injured tendons (hITPCs) were treated with vehicle (control) or rhBMP12 (BMP12, 100 ng/ml) in monolayer for 7 days. Expression of the tendon-related genes (*SCX*, Scleraxis; *TNMD*, Tenomoduline; *MKX*, Mohawk; and *COL1A1*, type 1 collagen α1) were examined by qPCR. (B and C) hITPCs were cultured in micromass and treated with 50 μg/ml ascorbic acid for 7 days followed by alcain blue staining. The staining intensity was quantified by ImageJ (C). D, Gene expression for chondrogenic (*SOX9* and *ACAN*) and tenogenic differentiation markers were compared between monolayer without rhBMP12 (Monolayer) and micromass cultures (Micromass). The Ct values of the qPCR analysis results were normalized to the *ACTB* Ct values, and the ratio to the control was calculated. The values are average +/- SD of the ratio to the control monolayer culture. n = 3 per group. *, p<0.05. The similar results were obtained from the experiments using other batches of hITPCs.

The cells isolated from human injured tendons were plated on a plastic culture dish at a low density, and the growing colonies were expanded. The passaged cultures were subjected to flow cytometric analysis to confirm that the cells expressed several typical mesenchymal stem cell markers including CD90, CD73 and CD105, but not hematopoietic markers as described previously (S1 Fig) [20]. To induce tenogenic differentiation, BMP12 was added into the monolayer culture [26,27]. The levels of gene expression of tenogenic differentiation markers, *SCX*, *TNMD* and *MKX* were increased 7 days after treatment compared to the vehicle-treated control culture (Fig 1A). When the cells were cultured in micromass, cultures accumulated matrix positive to alcian blue (Fig 1B and 1C) and increased gene expression levels of chondrogenic genes such as *SOX9* and *ACAN* while reduced those of *TNMD*, *MKX* and *COL1A* (Fig 1D). *SCX* was up-regulated in micromass culture. This may be due to an early change of skeletogenic differentiation in progenitors since *Scx* expression has been found in the sclerotome of the somite and mesenchymal precursors of axial, appendicular and craniofacial skeletal elements before the expression becomes specifically dominant in tendon [26]. These findings indicate that human injured tendon-derived cells, herein called hITPCs, have progenitor characteristics and potential to proceed toward chondrogenic and tenogenic differentiation.

### ^13^C-glucose metabolic flux analysis of hITPCs

**Fig 2:**
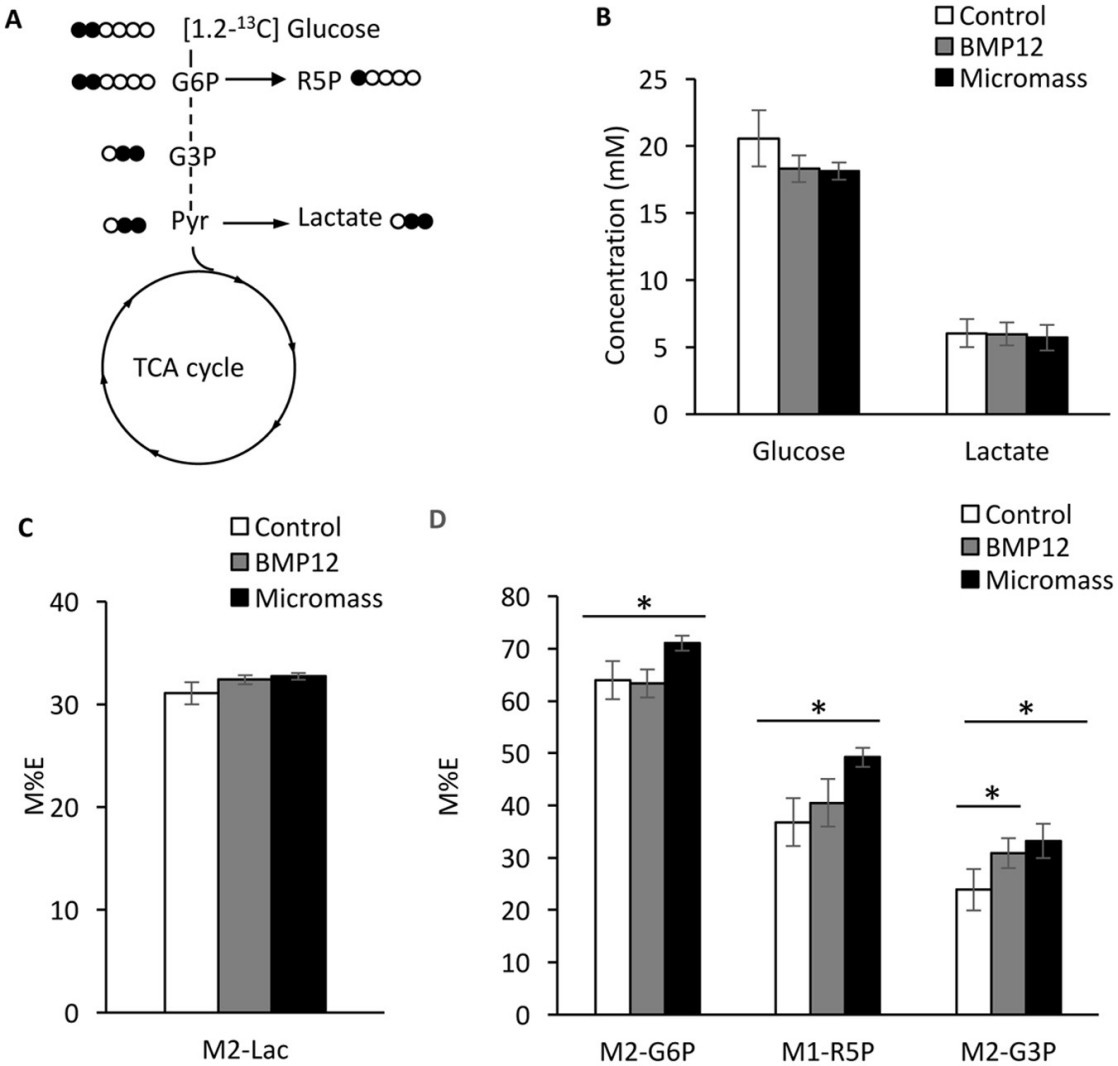
Flux of [1,2-^13^C] glucose to glycolysis, pentose phosphate and lactate synthesis pathways. The hITPCs were cultured in monolayer treated with vehicle (Control) or rhBMP12 (100ng/ml) (BMP12) or micromass (Micromass) cultures. After 6 days, the cultures were labeled [1,2-^13^C]-glucose for 48 hrs and lysed in 4% perchloric acid. The medium and acid-soluble fractions were subjected to metabolomics analysis. (A) Carbon fate map showing the isotopomer distribution of glucose-6-phosphate (G6P), glyceraldehyde-3-phosphate (G3P), pyruvate (Pyr), ribose-5 phosphate (R5P) and lactate derived from [1,2-^13^C]-glucose. (B) Concentration of glucose and lactate in the medium. (C) ^13^C-enrichment of M2-Lactate (M2-Lac) in the medium. (D) ^13^C-enrichment of G6P, R5P and G3P in the cell layer. The values are average +/- SD. n = 4 per group. *, p<0.05.

To investigate alteration of glucose metabolism when hITPCs proceed toward chondrogenic or tenogenic differentiation, we performed glucose metabolite flux analysis using [1,2-^13^C] glucose in the monolayer, BMP12-treated monolayer and micormass cultures, corresponding to the control, tenogenic and chondrogenic conditions. The isotopomer enrichment of the indicated metabolites derived from [1,2-^13^C] glucose showing in Fig 2A was analyzed. There is no significant difference between glucose consumption and lactate synthesis among the control, tenogenic (BMP12) and chondrogenic (Micromass) cultures (Fig 2B). ^13^C-enrichment of lactate M+2 did not show significant difference among these three groups either (Fig 2C). In contrast, the enrichment of ^13^C-labeled glucose-6-phosphate (G6P), ribose-5-phosphate (R5P) and glycelaldehyde-3-phosphate (G3P) were significantly increased in the micromass cultures (Fig 2D, Micromass). The enrichment of ^13^C-G3P was increased in tenogenic culture (Fig 2D, BMP12).

**Fig 3:**
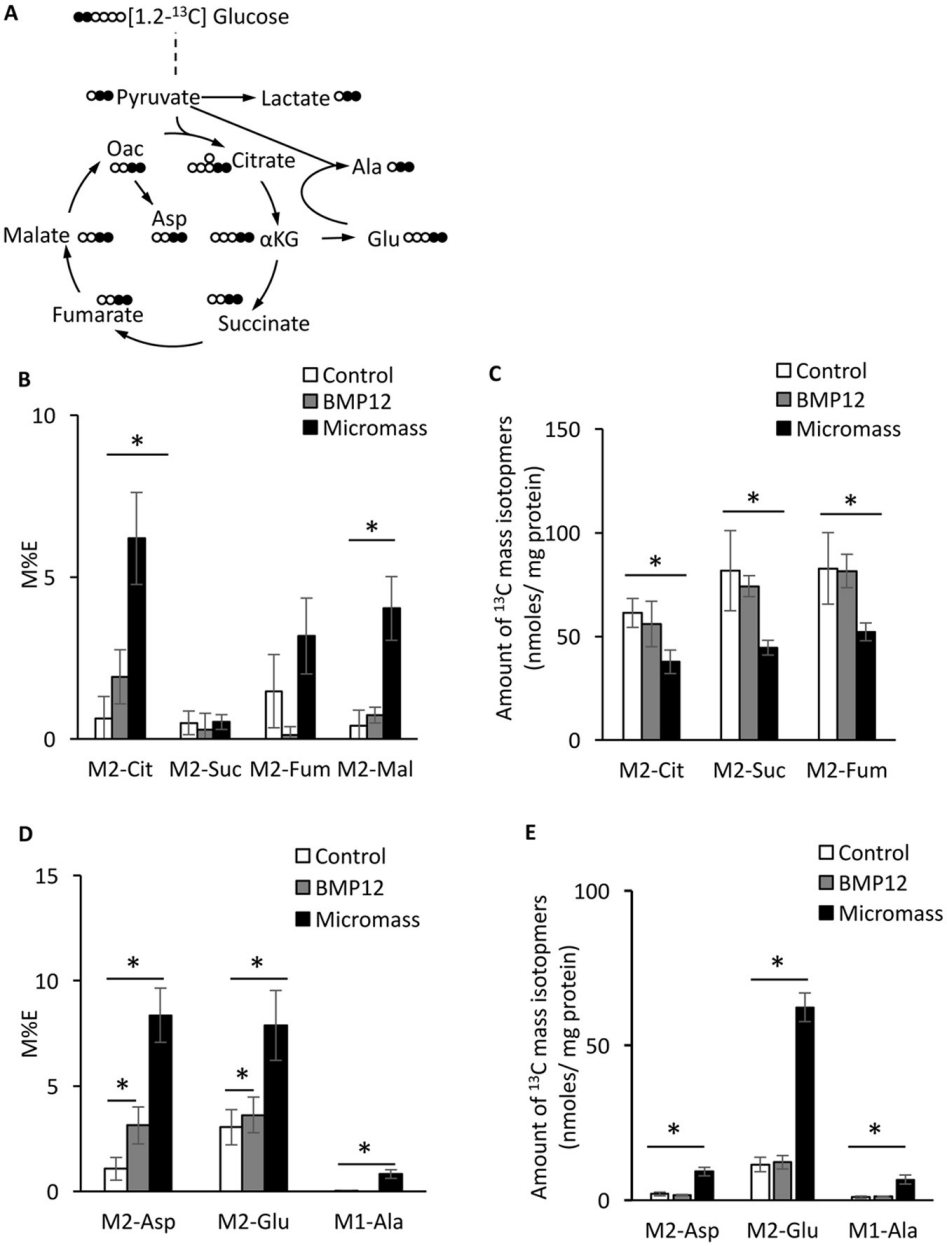
Flux of [1,2-^13^C] glucose to TCA cycle and amino acids derived from TCA cycle intermediates. The sample preparation is described in the legend of Fig 2. (A) Carbon fate map showing the isotopomer distribution of indicated metabolites, intermediates and amino acids derived from [1,2-^13^C] glucose. (B and C) ^13^C-enrichment (B) and amounts (C) of M2-citrate (M2-Cit), M2-succinate (M2-Suc), M2-fumarate (M2-Fum) and M2-malate (M2-Mal) in the cell layer. (D and E) ^13^C-enrichment (D) and amounts (E) of M2-aspartate (M2-Asp), M2-Glutamate (M2-Glu) and M2-alanine (M2-Ala) in the cell layer. The values are average +/- SD. n = 4 per group. *, p<0.05.

The enrichment of intermediates of TCA cycle -citrate, fumarate and malate- (Fig 3A) were increased in micromass culture (Fig 3B) whereas the amounts of these isotopomers were reduced (Fig 3C). It is suggested that these intermediates were rapidly used for biosynthesis of other molecules. Indeed, both enrichment and amount of the first products of amino acids from TCA cycle (Fig 3A) -aspartate (Asp), glutamate (Glu) and alanine (Ala)- were greatly increased (Fig 3D and 3E). The total amounts of amino acids were also increased in the chondrogenic cultures (S2 Fig). These findings indicate that hITPCs alter the glucose metabolic mode during they change their differentiation state, especially they undergo chondrogenic differentiation. Interestingly, we found increases in enrichment of M2-Asp and M2-Glu in BMP12-treated tenogenic culture, suggesting increases in TCA cycle activity and biosynthesis of amino acids when the hITPCs change their differentiation state toward tenogenic differentiation.

### The effects of 2-deoxy-D-glucose (2DG) on hITPCs in micromass cultures

**Fig 4:**
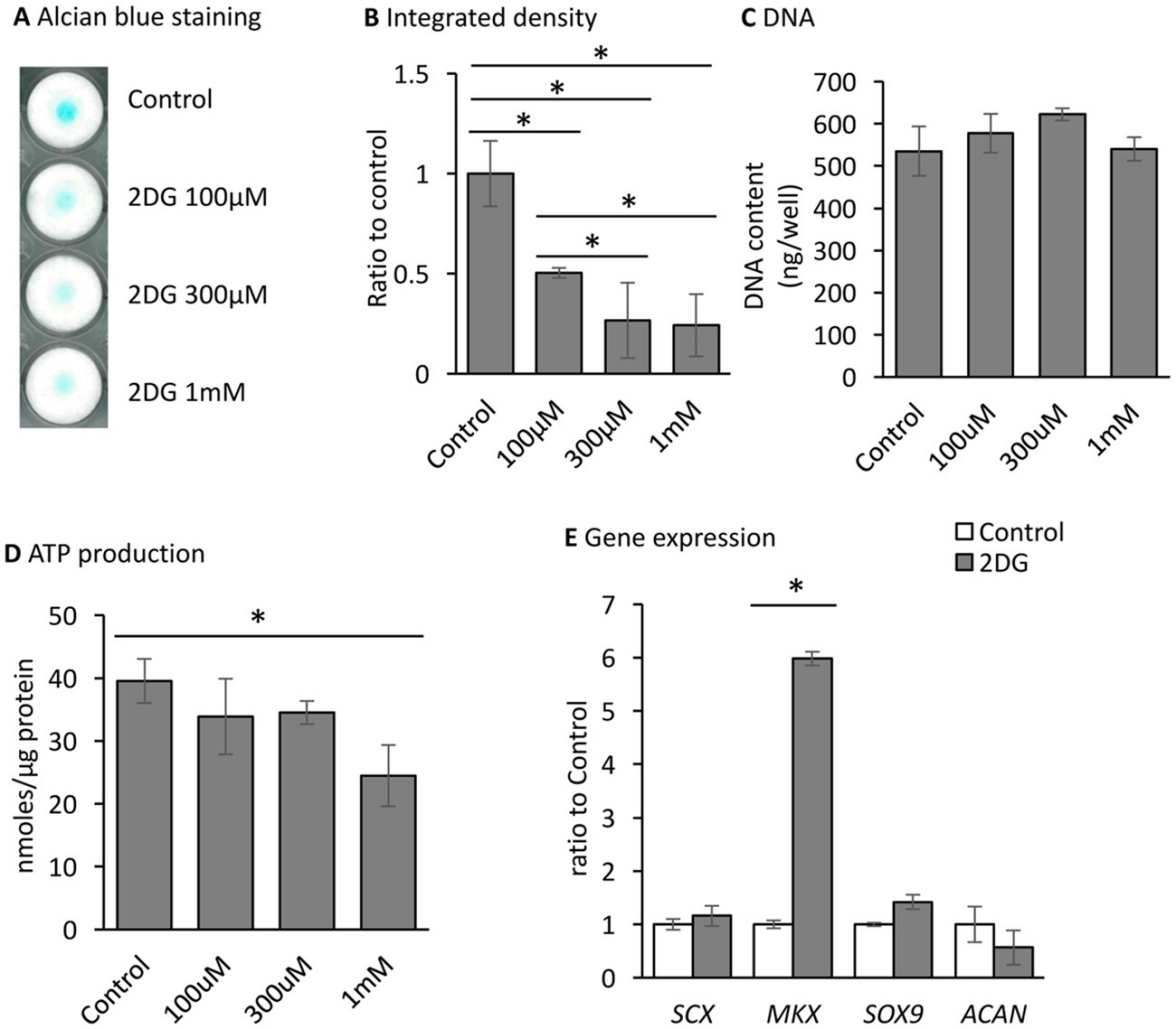
Inhibition of chondrogenic differentiation by 2DG. hITPCs were cultured in micromass and treated with vehicle or 2-deoxy-D-glucose (2DG) at the indicated concentrations for 7days. (A and B) the cultures were stained with alcian blue (A), and the integrated density of the staining result was measured (B). (C and D) the cultures were lysed containing 0.1% Triton-X and 0.01N NaOH. DNA (C) and ATP (D) contents were measured. E, gene expression of tenogenic (*SCX* and *MKX*) and chondrogenic (*SOX9* and *ACAN*) genes were examined by qPCR. The Ct values of the qPCR analysis results were normalized to the *ACTB* Ct values, and the ratio to the control was calculated. The values are average +/- SD. n = 3 per group. *, p<0.05.

To examine the significance of the increase in glycolysis, we used 2-deoxy-D-glucose (2DG) that is a glucose derivative and competitively inhibits Hexokinase, the enzyme of the first step of glycolysis [28]. Treatment with 2DG decreased the intensity of alcian blue staining in micromass culture (Fig 4A and 4B). The inhibition effect was found at 100 μM and reach a maximum at 300 μM. This effect was also found in the presence of a lower level of glucose (S3 Fig). The DNA contents were similar in the control and 2DG-treated cultures (Fig 4C), indicating that the decrease in alcian blue staining was not due to the inhibition of cell proliferation or cell death. 2DG treatment at the concentration of 1 mM mildly but significantly reduced ATP production (Fig 4D). Interestingly, the expression level of *MKX* was greatly stimulated by 2DG treatment (Fig 4E).

### Effects of 2DG on hITPCs in monolayer culture

**Fig 5:**
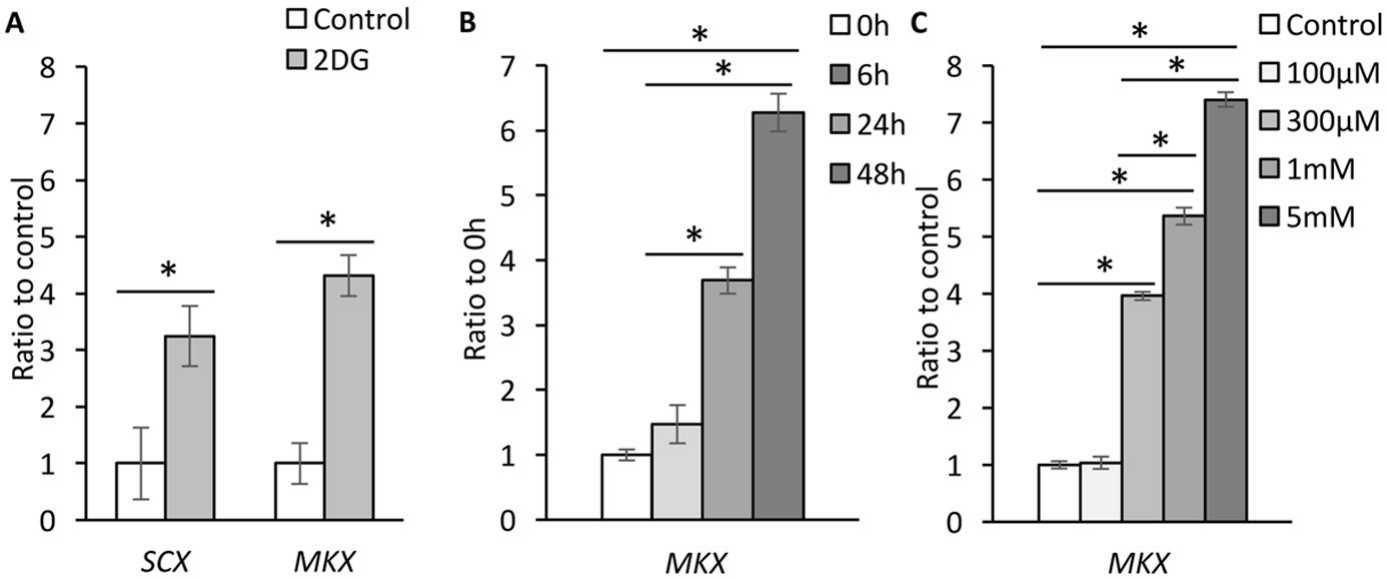
Stimulation of *MKX* gene expression by 2DG. hITPCs were cultured in monolayer and treated with vehicle or 2-deoxy-D-glucose (2DG, 1 mM for A and B, or indicated concentration for C in the presence of rhBMP12 (100ng/ml) at the indicated concentrations for 7days (A), indicated period (B) or for 48 hrs (C). Gene expression of *SCX* and/or *MKX* was examined by qPCR. The Ct values of the qPCR analysis results were normalized to the *ACTB* Ct values. The values are average +/- SD. n = 3 per group. *, p<0.05.

The stimulatory action of 2DG on *MKX* gene expression was also found in monolayer culture. 2DG treatment stimulated gene expression levels of *SCX* as well as *MKX* (Fig 5A). Up-regulation of *MKX* gene expression was detectable 6 h after treatment and significant as early as 24h after treatment (Fig 5B) and with 300 μM or higher concentration of 2DG (Fig 5C). The effects of 2DG on *MKX* gene expression and GAG accumulation were also found in low glucose medium (S3 Fig).

## Discussion

The results in this study indicate that the human injured tendon-derived progenitor cells change glucose metabolism when they proceed toward tenogenic or chondrogenic differentiation. Furthermore, our findings demonstrate that 2DG, an inhibitor of glycolysis stimulated gene expression of tenogenic transcription factors in these cells. The findings suggest that control of glucose metabolism would be beneficial for tenogenic differentiation of connective tissue progenitors. There are limitations in this study. The study was performed using progenitor cells isolated from injured flexor tendons. Therefore, interpretation of the results should be limited to this specific cell fraction at this moment. We should extend the study to different types of tendon progenitors obtained from normal tendons and other conditioned tendons. We used monolayer for stimulation of tenogenic differentiation to secure enough materials for the metabolomics analysis. Although BMP12 stimulated gene expression of several tenogenic markers, use of 3D culture may provide clearer metabolic changes associated with tenogenic differentiation. Similarly, we should test other chondrogenic conditions such as with chondrogenic factors and 3D pellet cultures. The significance of glucose control by 2DG in tendon repair need to be tested in vivo.

### Changes in glucose metabolism

Recent studies have demonstrated that glucose metabolism is altered in injured and healing tendons in human and mice. The changes in glucose metabolism include increases in glucose, lactate and pyruvate contents in the human healing Achilles tendons [9] and stimulation of glycolysis, lactate synthesis and TCA cycle in mouse injured Achilles tendons [10]. These studies have not shown which cells cause the metabolic changes. The results in this study indicate that the hITPCs slightly stimulated glycolysis activity and biosynthesis of amino acids from the intermediates of TCA cycle when they expressed tenogenic phenotype in response to BMP12. The hITPCs strongly stimulated TCA pathway and biosynthesis of amino acids when they were cultured in micromass, suggesting that progenitors appearing in injured tendons are one of the cells involving in the metabolic changes detected in the healing and injured tendons. This assumption is also supported by previous findings that stem cells and progenitors reprogram glucose metabolism and energy state during their differentiation processes [1, 27].

The significance of metabolic changes in tendon healing has remained unclear. Elevation of glucose and glucose metabolites in early healing tendons may support tendon healing since the intermittent pneumatic compression that experimentally stimulates tendon repair further increases concentrations of these molecules [9]. In contrast, inhibition of lactate synthesis in injured tendons has improved recovery of collagen fiber alignment and mechanical properties at the early healing phase, suggesting that an increase in lactate synthesis may disturb tendon healing [10]. The damaged tendons often show cartilaginous metaplasia characterized by mucoid accumulation and up-regulation of chondrogenic genes such as *Aggrecan* and *type 2 collagen* [28, 29]. This pathology may be caused by dysregulation of tendon cells or mis-differentiation of tendon progenitors [18, 20, 30]. The changes in glucose metabolism in the hITPCs observed in this study may be coupled with cartilaginous metaplasia in damaged tendons. The hITPCs greatly changed their glucose metabolic status in micromass culture without addition of pro-chondrogenic factors such as TGFβs and BMP2. This metabolic alteration may be linked to a change in cytoarchitecture or mechanical force.

### 2DG on chondrogenesis

Glycolysis is generally reduced in stem/progenitor cells during differentiation [1]. This is not in the case of chondrogenic differentiation of mesenchymal progenitors. Up-regulation of Hif-1a is found in the core of the developing cartilage [31, 32], which may be a result from hypoxia. Since Hif-1a is a key transcription factor that stimulates gene expression of several key enzymes for glycolysis [33], the glycolysis is expected to increase in cartilage although the conclusive evidence is not available to directly demonstrate an increase in glycolysis in the developing cartilage *in vivo*. Studies have demonstrated that hypoxic condition support chondrogenic differentiation in mesenchymal stem cells [34]. Furthermore, a high-glucose diet increases cartilaginous lesion in injured rat Achilles tendons [35]. Hence, restriction of glycolysis may be effective to inhibit mis-differentiation of tendon progenitors in injured sites as we demonstrated chondrogenic inhibition by 2DG. 2DG competitively inhibits hexokinases [36] that are the limited enzymes of the glycolysis pathway. Inhibition of glycolysis leads to inhibition of ATP production. Control of concentration and oscillation of ATP is important for chondrogenesis and chondrocyte differentiation [37, 38]. However, the dosage of 2DG that showed inhibition of ATP production was much higher than the one that inhibited proteoglycan accumulation, suggesting that the mechanism of the inhibition by 2DG may not be due to inhibition of ATP production. 2DG might reduce the concentrations of precursors for proteins since inhibition of glycolysis likely reduces TCA cycle activity and biosynthesis of amino acids. Indeed, a high demand for amino acids in chondrocytes has been reported [39], and the contents of most amino acids increased in chondrogenic hITPC cultures. In addition, 2DG may directly inhibit to produce precursors of glycosaminoglycans by blocking generation of glucose-6-phosphate from glucose [36]. Treatment with 2DG induces de-differentiation of rabbit articular chondrocytes [40]. This study indicates that the action of 2DG is via inhibition of GSK3β, leading to stimulation of Wnt/β-catenin signaling. The same mechanism may work in the hITPC cultures.

### 2DG on expression of tenogenic transcription factors

High glucose gives negative impact on tendon homeostasis. Clinical data has shown a clear relation between hyperglycemia and tendon disorders [11–14]. Experimentally, high glucose disturbs tendon repair in rat Achilles tendons [35] and decreased gene expression of tendon-associated genes including *Mkx* in rat Achilles tendon cells in culture [41] and rat tendon stem/progenitors [21]. In our culture system, we did not see down-regulation of *MKX* and *SCX* gene expression in the high glucose medium (data not shown). However, 2DG treatment significantly stimulated the expression of these genes. In particular, *MKX* gene greatly and rapidly responded to 2DG treatment. These results suggest that limiting glucose use is favorable for tenogenic condition although further studies on the actions of 2DG on tenogenesis are required. The MKX [42, 43] as well as SCX [44] have been identified as essential transcription factors in tendon development. While *Scx*-deficiency induces defects in tendon formation [45], *Mkx*-deficiency leads to defects in tendon tissue maturation [43] and heterotopic ossification in rodents [46, 47]. Interestingly, *Mkx* null tendon-derived cells have stronger potential to induce chondrogenic differentiation while overexpression of *Mkx* inhibits chondrogenic differentiation [46]. Our results that 2DG stimulated *MKX* expression and inhibited chondrogenic differentiation are consistent with the phenotype seen in loss- and gain-of-*Mkx* function studies. Kayama et al. [48] have demonstrated that a general transcription factor II-I repeat domain-containing protein 1 (*Gtf2ird1*) is important for transcription of *Mkx* in response to mechanical force. Interestingly, *Gtf2ird1* has been shown to play an important factor in the molecular network controlling glucose homeostasis [49]. The mechanism underlying stimulation of *MKX* by 2DG needs to be elucidated in future.

In conclusion, glucose metabolism was altered in human injured tendon derived progenitors when they proceed toward tenogenic or chondrogenic differentiation. The results suggest that inhibition of glucose use disturbs chondrogenic differentiation and stimulates tenogenic differentiation in these cells.

## Supporting information

S1 Fig Expression of stem cell markers in hITPCs. The hITPCs are harvested from the monolayer culture and subjected to flow cytometry analysis. The hIPTC expressed stem cell markers, CD73, CD90 and CD105, but not hematopoietic stem cell markers (CD45, CD34, CD11b, CD79a and HLA-DR). Similar results were obtained from all batches examined.
(TIF)

S2 Fig The amounts of amino acids in the medium of the hITPC monolayer or micromass culture. The hITPCs were cultured in monolayer treated with vehicle (Control) or rhBMP12 (100ng/ml) (BMP12) or micromass (Micromass) cultures. After 6 days, the cultures were labeled [1,2-^13^C]-glucose for 48 hrs and lysed in 4% perchloric acid. The total amounts of indicated amino acids in the cell layer were measured.
(TIF)

S3 Fig The effects of 2DG on hITPCs cultured in low glucose medium. The hITPCs isolated from the injured flexor tendon were cultured in micromass (Alcian blue staining) and monolayer (MXK expression) cultures in high glucose (4.5 g/L) or low glucose (1.0 g/L) DMEM. The cultures were treated with 100 μM 2DG in low glucose DMEM for 7 days. The cultures (n = 3) were subjected to Alcian blue staining or qPCR to examine *MKX* gene expression.
(TIF)
