# Supplementary figures and images for: Control of glucose metabolism is important in tenogenic differentiation of progenitors derived from human injured tendons
Source: PLoS One. 2019 Mar 18;14(3):e0213912. doi: 10.1371/journal.pone.0213912 (PMC6422258; doi:10.1371/journal.pone.0213912)

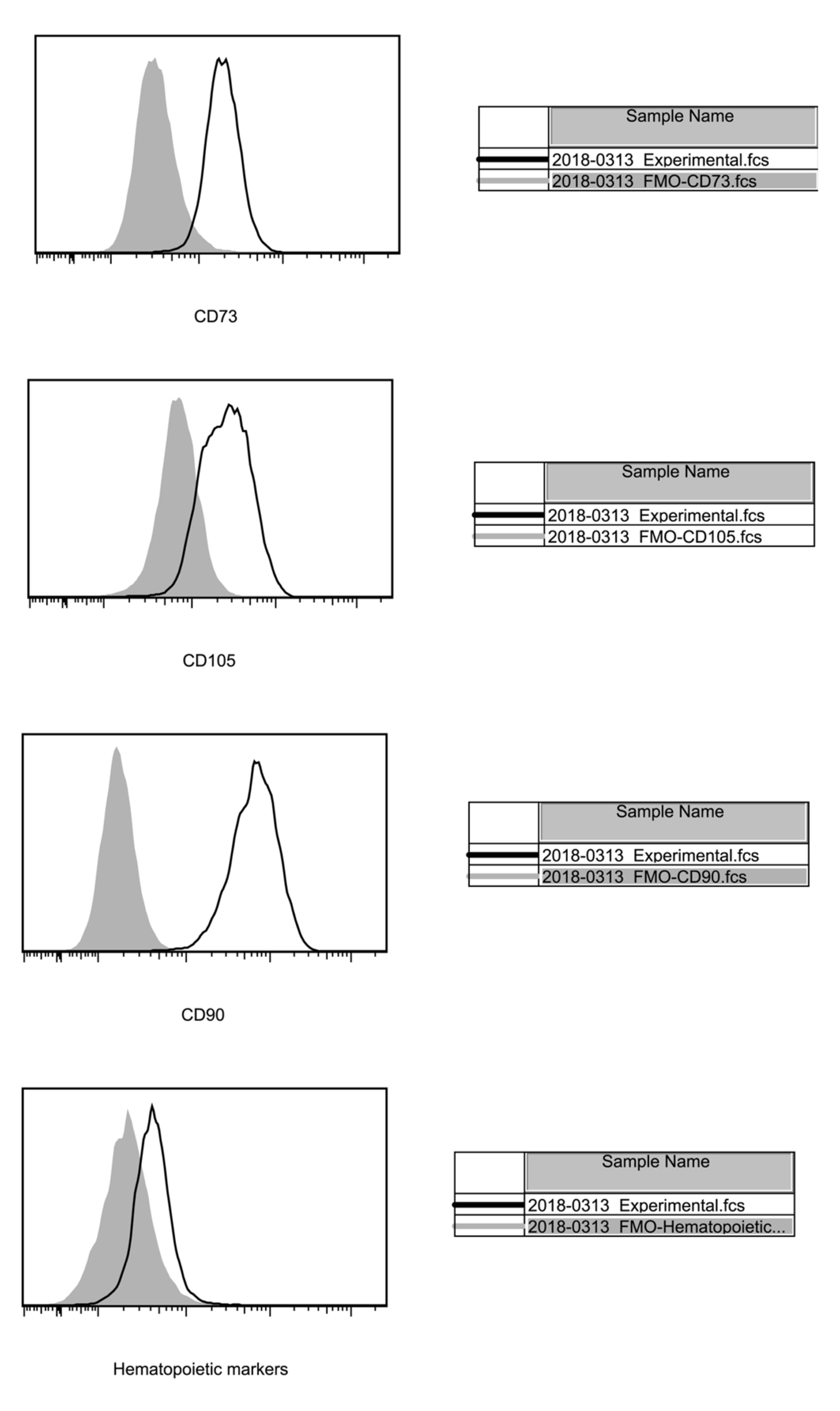

Supplement: S1 Fig — The hITPCs are harvested from the monolayer culture and subjected to flow cytometry analysis. The hIPTC expressed stem cell markers, CD73, CD90 and CD105, but not hematopoietic stem cell markers (CD45, CD34, CD11b, CD79a and HLA-DR). Similar results were obtained from all batches examined. (TIF) [file pone.0213912.s001.tif]

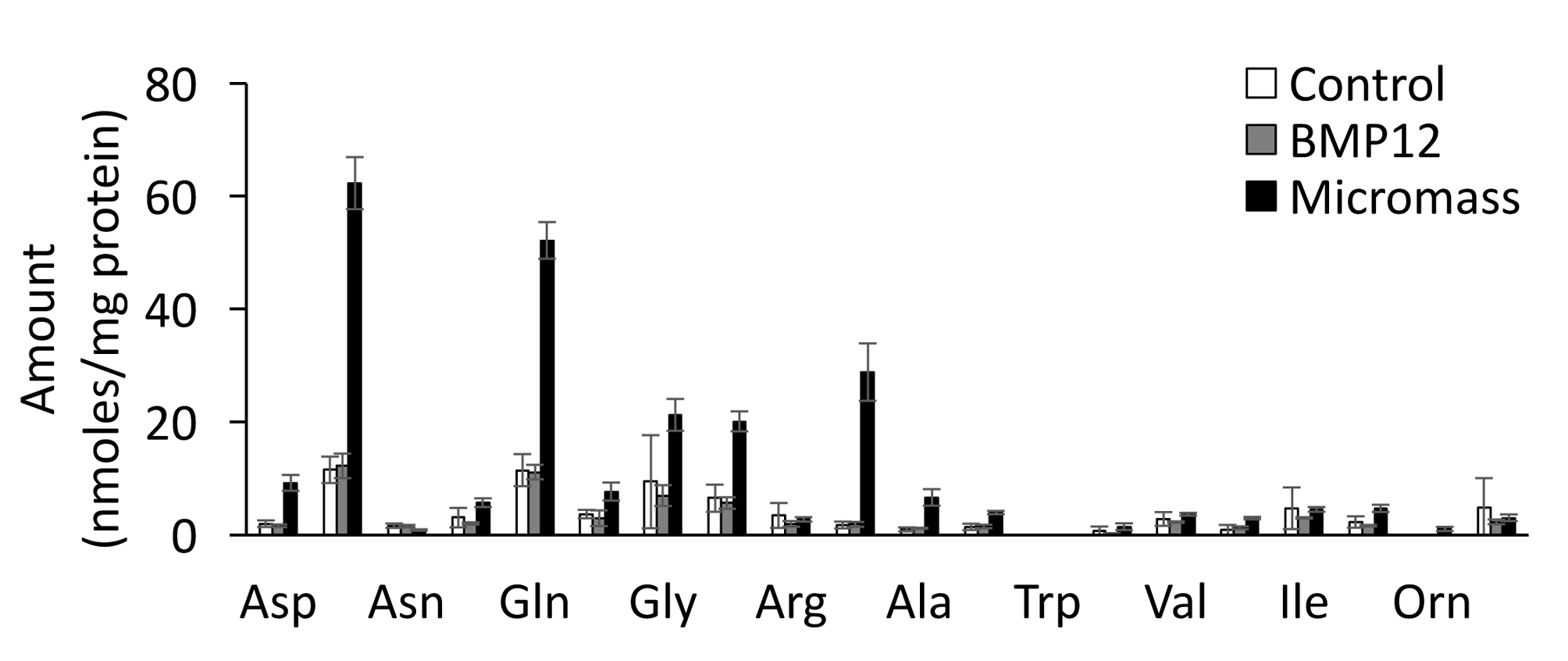

Supplement: S2 Fig — The hITPCs were cultured in monolayer treated with vehicle (Control) or rhBMP12 (100ng/ml) (BMP12) or micromass (Micromass) cultures. After 6 days, the cultures were labeled [1,2-13C]-glucose for 48 hrs and lysed in 4% perchloric acid. The total amounts of indicated amino acids in the cell layer were measured. (TIF) [file pone.0213912.s002.tif]

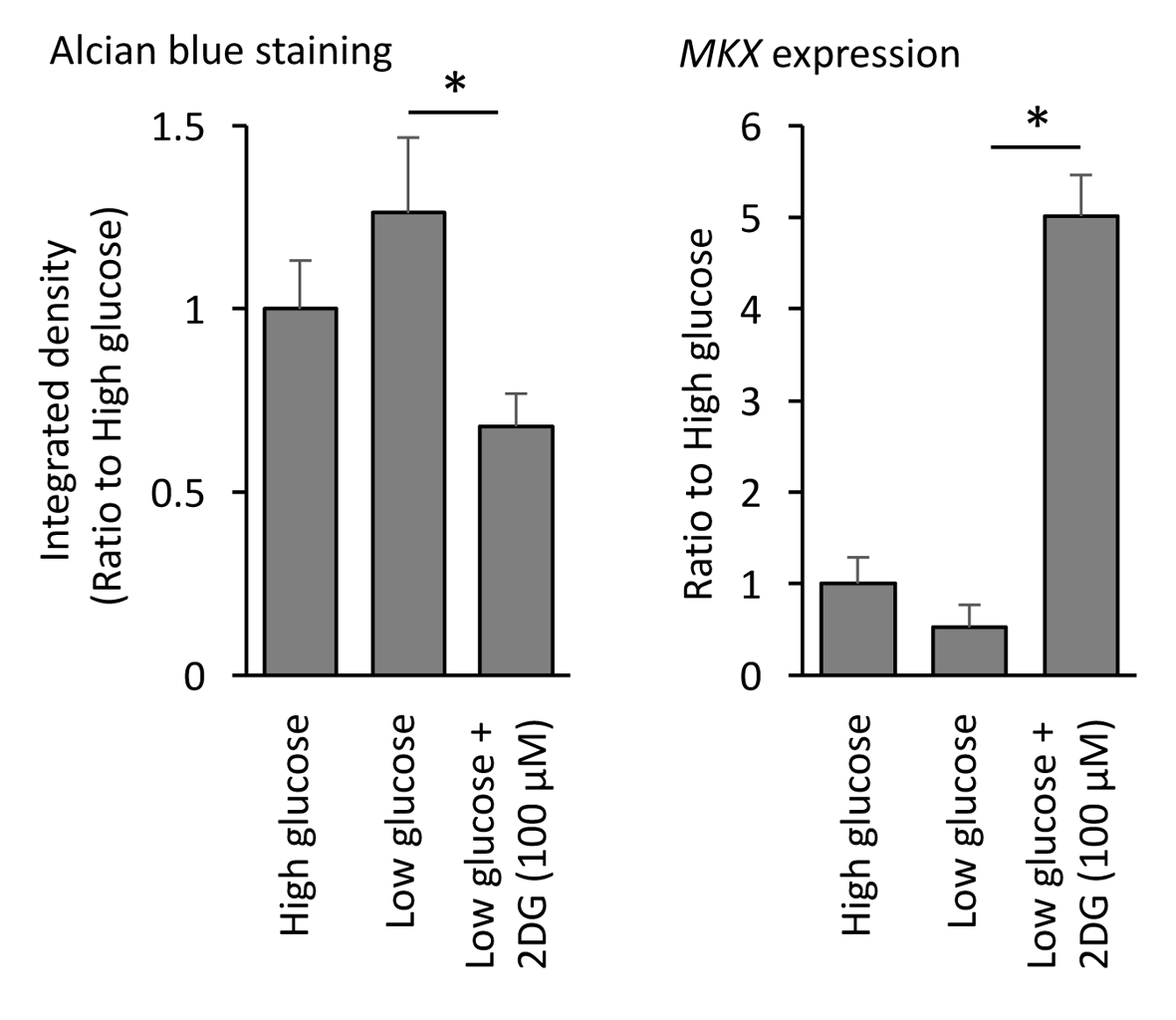

Supplement: S3 Fig — The hITPCs isolated from the injured flexor tendon were cultured in micromass (Alcian blue staining) and monolayer (MXK expression) cultures in high glucose (4.5 g/L) or low glucose (1.0 g/L) DMEM. The cultures were treated with 100 μM 2DG in low glucose DMEM for 7 days. The cultures (n = 3) were subjected to Alcian blue staining or qPCR to examine MKX gene expression. (TIF) [file pone.0213912.s003.tif]
